# Treatment outcomes of posterior pilon fractures using a simple single lateral approach *via* stretching fibular fracture line

**DOI:** 10.3389/fsurg.2023.1141606

**Published:** 2023-03-30

**Authors:** Wei Liang, Mingping Zhou, Zhongting Jiang, Xuanyu Mao, Xiang Zhou

**Affiliations:** ^1^ Department of Orthopaedics, Lishui People's Hospital, The Sixth Affiliated Hospital of Wenzhou Medical University, The First Affiliated Hospital of Lishui University, Lishui, China; ^2^ Department of Orthopaedics, Longquan People’s Hospital, Longquan, China

**Keywords:** posterior pilon fracture, single lateral approach, posterolateral approach, fibular fracture line, treatment outcome

## Abstract

**Purpose:**

The aim of this study was to explore the treatment outcomes of a novel single lateral approach *via* fibular fracture line for patients with posterior pilon fractures.

**Patients and methods:**

From January 2020 to December 2021, a total of 41 patients with posterior pilon fractures who received surgical treatment in our hospital were retrospectively reviewed. Twenty patients (Group A) were treated with open reduction and internal fixation (ORIF) *via* posterolateral approach. Twenty-one patients (Group B) were treated with ORIF using a simple single lateral approach *via* stretching fibular fracture line. Clinical assessments, including operation time, intraoperative blood loss, the American Orthopaedic Foot and Ankle Society (AOFAS) ankle-hindfoot score, visual analogue scale (VAS), and the active range of motion (ROM) of the ankle at the final follow-up visit after surgery, were performed in all patients. Radiographic outcome was evaluated by using the criteria proposed by Burwell and Charnley.

**Results:**

The mean follow-up time was 21 months (range 12–35). The average operation time and intraoperative blood loss in the Group B were significantly less than those in the Group A. Moreover, the AOFAS score and ankle ROM in the Group B were significantly higher than those in the Group A at the final follow-up visit. Eighteen cases (90%) in Group A and 19 cases (90.5%) in Group B achieved anatomical reduction of the fracture.

**Conclusion:**

The single lateral approach *via* stretching fibular fracture line is a simple and effective technique for reduction and fixation of posterior pilon fractures.

## Introduction

Posterior articular fractures of the distal tibia include posterior malleolus fractures and posterior pilon fractures (1). Previous studies showed that posterior pilon fractures have a low incidence and variation in fracture morphology. Recently, posterior pilon fractures has gained increasing interest in clinical practice due to its poor outcome (2, 3). Anatomically, posterior pilon fractures extends to the medial malleolus (4, 5). Posterior pilon fractures are caused by rotation and axial load and characterized with large fracture fragments (6). Meanwhile, the treatment for this fracture type has been increasingly reported and remains a challenge. However, the best surgical approach for posterior pilon fractures has not been determined (7).

Currently, a single incisional posterolateral approach is often chosen for treating posterior pilon fractures (1). But this approach cannot fully expose the broken end of the bone and the articular surface of the distal tibia. In order to achieve clear visualization of the surgical field and minimal operative trauma, we innovatively used a simple single lateral approach *via* stretching fibular fracture line to treat posterior pilon fractures combined with a fibula fracture at the same level. The aim of the study was to evaluate the clinical efficacy of this surgical approach and promote its clinical application.

## Material and methods

### Study population

A retrospective analysis in 41 patients with posterior pilon fractures treated between January 2020 and December 2021 was conducted. Twenty patients (Group A) were treated with open reduction and internal fixation (ORIF) *via* posterolateral approach. Twenty-one patients (Group B) were treated with ORIF using a simple single lateral approach *via* stretching fibular fracture line. All surgeries were performed by the same team. This research was approved by the Ethics Committee of Lishui City People's Hospital and was in accordance with the Helsinki Declaration. All patients gave informed consent and signed informed consent.

Our inclusion criteria were (1) posterior pilon fractures combined with fibula fracture at the same level (2). Those who were treated with ORIF *via* posterolateral approach or lateral approach *via* stretching fibular fracture line. Our exclusion criteria were (1) Pathological fractures and open fractures (2) Those who cannot tolerate surgery (3) Follow-up data were incomplete and the follow-up time was less than 12 months.

### Surgical approach

**Figure 1:**
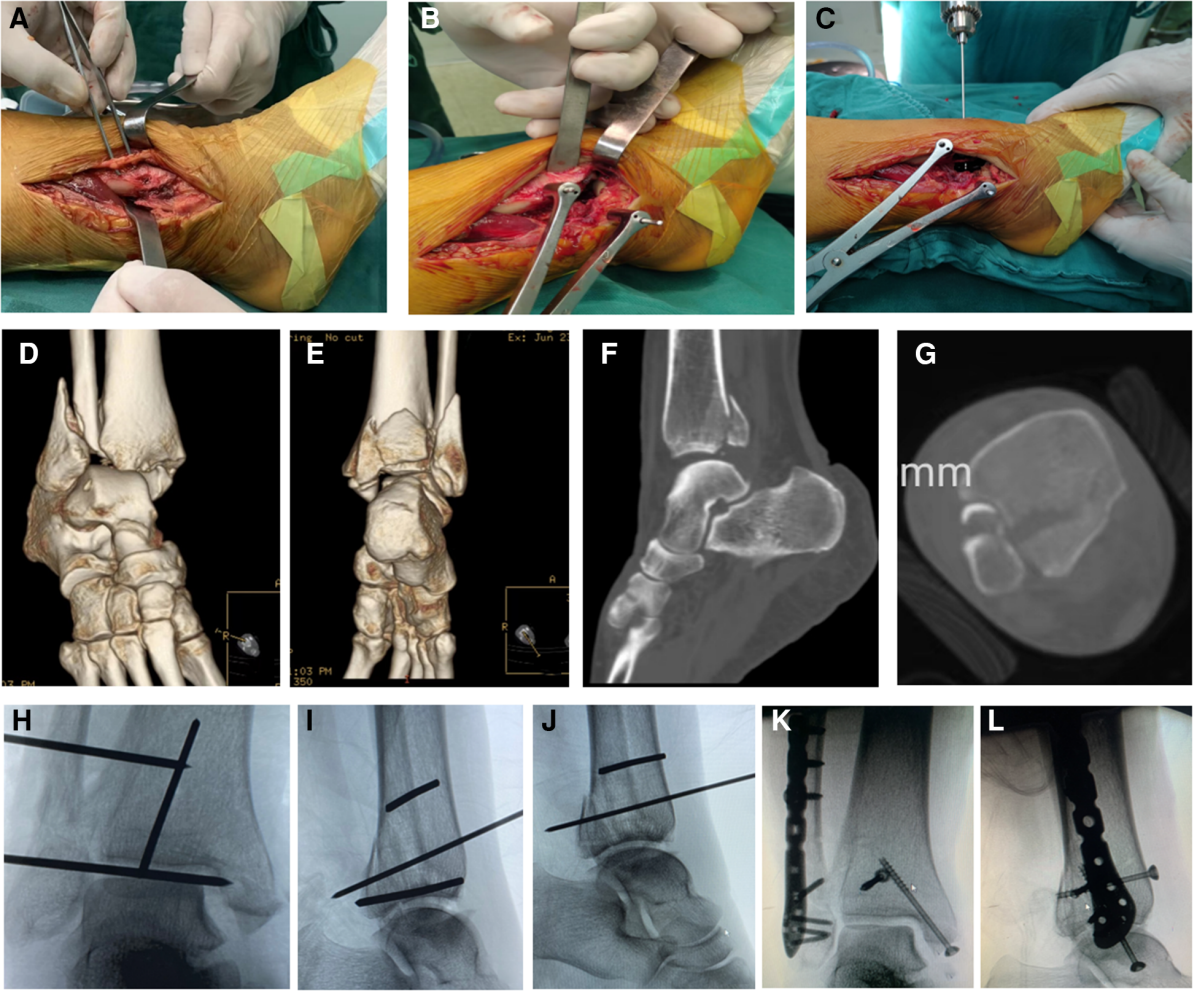
Surgical procedure of a single lateral approach *via* stretching fibular fracture line. (**A**) A single lateral incision was used to expose the broken end of the fibula fracture. (**B**) The ankle joint was distracted with a Hintermann retractor to obtain sufficient working space. (**C**) A hollow guide needle was inserted from the anterior tibia to the posterolateral tibia to fix the posterior malleolus fracture. (**D,E**) Preoperative 3D reconstruction images. (**F**) Preoperative sagittal CT image. (**G**) Preoperative transverse CT image. (**H**) Intraoperative anterior–posterior x-ray image showed the fixation of the fracture with Kirschner wires. (**I**) Intraoperative lateral x-ray image showed the fixation of the posterior malleolus fracture with the Kirschner wire and the fracture reduction was mildly poor. (**J**) After adjustment, anatomic reduction with the Kirschner wire. (**K**) Postoperative anterior–posterior x-ray image. (**L**) Postoperative lateral x-ray image.

In the Group A, posterolateral approach was used for osteosynthesis of the posterior and lateral malleolus (8). In the Group B, we used a simple single lateral approach *via* stretching fibular fracture line to treat both posterior pilon fractures and fibular fractures (Figure 1). Two 2.0 mm Kirschner wires were placed at both ends of the fibula fracture line and inserted into both fibula and tibia. Then the Hintermann retractor was used to open the gap between the fibula fractures. Thus, the broken end of the tibia can be fully exposed. Surgeons can observe the articular surface of the distal tibia under direct vision and reduce and fix the posterior malleolus fracture block under direct vision. Ultimately, reduction and internal fixation of the fibula fracture was performed. Notably, this approach has less interference with the surrounding soft tissue. Conclusively, our novel approach allows for minimally invasive reduction and firm fixation.

### Assessments

All patients were followed up for over 12 months. Basic clinical information, operating time, and intraoperative blood loss were recorded. The time to fracture healing and the time to full bear weight were documented during the follow-up. The American Orthopaedic Foot and Ankle Society (AOFAS) ankle-hindfoot score, visual analogue scale (VAS), the active range of motion (ROM) of the ankle, and complications were recorded at regular follow-up after surgery. The radiological outcome was evaluated through standard XR using the criteria proposed by Burwell and Charnley (9).

### Statistical methods

All statistical analyses were performed by using IBM SPSS Statistics 22.

Quantitative variables were shown in the form of mean ± standard deviation and a *t*-test was used for the comparison. Categorical variables were presented in the form of frequency (%) and a *χ*^2^ test and Fisher exact test were used for analyses. Variables with two-tailed *p* < 0.05 were considered statistically significant.

## Results

### Patient characteristics

There were 26 male and 15 female included in the study, aged 28–60 years (average age 44.6 years). Five patients were excluded, including two open fractures, two lost to follow-up, and one with less than 1 year of follow-up. The average BMI of all patients was 23.9 kg/cm^2^ (20.7–28.3). According to Lauge–Hansen classification, there were 17 cases of supination-external rotation (SER) type III ankle fracture, 24 of SER type IV ankle fracture. The causes of injury were falling from height (*n* = 17), twist (*n* = 7), and traffic accident (*n* = 17). The postoperative follow-up duration was 12–35 months (mean 21months). There was no significant difference between the two groups in age, gender, BMI, Lauge–Hansen classification, follow-up, and cause of injury (*p* > 0.05) (Table 1).

### Clinical outcomes

Group A had significantly more operation time and intraoperative blood loss than group B (*p* < 0.001) (Table 2). In terms of fracture healing time and full weight-bearing time, no significance was observed between the two groups (Table 3). Additionally, there was no significance in VAS between the two groups (Table 3). The mean AOFAS and ankle ROM in Group B was significantly higher than that in Group A at the final follow-up visit (*p *< 0.05) (Table 3).

**Table 1:** Patient demographics.

	All (*n* = 41)	Group A (*n* = 20)	Group B (*n* = 21)	*p*-value
**Mean age, years**	44.6 ± 8.4	44.2 ± 8.7	45 ± 8.3	0.75
**Gender, *n* (%)**				0.658
Male	26 (63.4%)	12 (60%)	14 (66.7%)	
Female	15 (36.6%)	8 (40%)	7 (33.3%)	
**BMI, kg/cm^2^**	23.9 ± 1.7	23.7 ± 1.8	24.1 ± 1.7	0.399
**Lauge-Hansen classification**				0.654
SER type III	17 (41.5%)	9 (45%)	8 (38.1%)	
SER type IV	24 (58.5%)	11 (55%)	13 (61.9%)	
**Follow-up, months**	21.0 ± 7.3	20.9 ± 8.3	21.2 ± 6.4	0.884
**Cause of injury, *n* (%)**				0.889
Fall from height	17 (41.5%)	9 (45%)	8 (38.1%)	
Twist	7 (17.1%)	3 (15%)	4 (19%)	
Traffic accidents	17 (41.5%)	8(40%)	9(42.9%)	

Mean ± standard deviations for the two groups.

**Table 2:** Operation time and intraoperative blood loss.

	Group A (*n* = 20)	Group B (*n* = 21)	*p*-value
Operation time, min	93.5 ± 6.8	60.5 ± 4.8	**<0** **.** **001**
Intraoperative blood loss, ml	51.5 ± 12.4	20.5 ± 4.4	**<0** **.** **001**

Mean ± standard deviations for the two groups. Bold indicates statistically significant.

**Table 3:** Clinical and radiographic outcomes of the two groups.

	Group A (*n* = 20)	Group B (*n* = 21)	*p*-value
Fracture healing time, month	3.1 ± 0.4	2.9 ± 0.3	0.115
Full weight-bearing time, month	3.2 ± 0.3	3.1 ± 0.2	0.242
AOFAS	86.6 ± 7.1	89.6 ± 7.2	**0** **.** **022**
VAS	0.9 ± 0.7	0.8 ± 0.5	0.644
Ankle ROM, degree	58.4 ± 2.8	61.3 ± 3.9	**0** **.** **007**

Mean ± standard deviations for the two groups. Bold indicates statistically significant.

According to the postoperative radiological examination, 18 cases (90%) in Group A and 19 cases (90.5%) in Group B achieved anatomical reduction of the fracture. Wound healing was satisfactory in all patients. No incision infection, deep vein thrombosis, and sural nerve injury occurred. No internal fixation failure and bone ununion were observed during the follow-up.

## Discussion

In clinical practice, several approaches for posterior pilon fracture fixation have been reported (10–13). However, optimal approaches for minimizing the operative trauma and achieve effective internal fixation has not yet been established. In order to achieve satisfactory articular function outcomes, we innovatively used a simple single lateral approach *via* stretching fibular fracture line to treat posterior pilon fractures combined with a fibula fracture at the same level. This approach has the advantages of clear visualization of the surgical field and less soft tissue interference. Notably, when posterior pilon fractures are not accompanied by fibula fracture, this approach is not appropriate for treatment. To our knowledge, this is the first research that compared the clinical effect of posterior pilon fractures between the single lateral approach *via* stretching fibular fracture line and the posterolateral approach.

According to our results, the operation time in those who were treated with a single lateral approach *via* stretching fibular fracture line was shorter and the intraoperative blood loss in those patients was also less. This is mainly because application of a single lateral approach *via* stretching fibular fracture line can fully expose the articular surface of the distal tibia and collapsed articular surface with less soft tissue interference. Furthermore, posterior pilon fractures can be reduced and fixed under direct vision with less soft tissue interference.

The average AOFAS score was 86.6 points in Group A, which was similar with some previous studies (1, 4, 10). Moreover, Group B had more satisfactory clinical outcomes than Group A in terms of AOFAS and ankle ROM, suggesting the advantages of the lateral approach *via* stretching fibular fracture line in postoperative functional recovery. Additionally, high anatomical reduction rate (90.5%) was achieved by using this approach. This is mainly because the surgical approach gives full play to the function of the Hintermann retractor's distraction and reduction. Some studies applied a similar method-the transfibular approach for posterior malleolus fracture fixation (14–16). However, these studies did not use retractors for distraction and reduction, which may make anatomic reduction difficult. The results of our study suggest that a single lateral approach *via* stretching fibular fracture line for the treatment of posterior pilon fractures can achieve good clinical efficacy, which is worthy of clinical promotion and application.

Notably, there are some limitations presented in this study. First, the retrospective nature of this study can lead to bias. Second, the sample size included in this study was relatively small. Relevant clinical studies with larger sample sizes can be carried out in the future. Additionally, future multi-center, prospective, randomized controlled studies should be conducted to further confirm the clinical value of our innovative surgical approach. Nevertheless, this study lays a foundation for subsequent clinical research on posterior pilon fractures.

## Conclusion

For posterior pilon fractures combined with fibula fractures at the same level, a simple single lateral approach *via* stretching fibular fracture line has good clinical effect and prognosis, which provides an alternative for the treatment of this kind of fracture.

## Data Availability

The raw data supporting the conclusions of this article will be made available by the authors, without undue reservation.
